# Crystal structure of *Bacillus cereus* flagellin and structure-guided fusion-protein designs

**DOI:** 10.1038/s41598-018-24254-w

**Published:** 2018-04-11

**Authors:** Meong Il Kim, Choongdeok Lee, Jaewan Park, Bo-Young Jeon, Minsun Hong

**Affiliations:** 1 Division of Biological Science and Technology, Yonsei University, Wonju, 26493 Republic of Korea; 2 Department of Biomedical Laboratory Science, Yonsei University, Wonju, 26493 Republic of Korea

## Abstract

Flagellin is a major component of the flagellar filament. Flagellin also functions as a specific ligand that stimulates innate immunity through direct interaction with Toll-like receptor 5 (TLR5) in the host. Because flagellin activates the immune response, it has been of interest to develop as a vaccine adjuvant in subunit vaccines or antigen fusion vaccines. Despite the widespread application of flagellin fusion in preventing infectious diseases, flagellin-antigen fusion designs have never been biophysically and structurally characterized. Moreover, flagellin from *Salmonella* species has been used extensively despite containing hypervariable regions not required for TLR5 that can cause an unexpected immune response. In this study, flagellin from *Bacillus cereus* (BcFlg) was identified as the smallest flagellin molecule containing only the conserved TLR5-activating D0 and D1 domains. The crystal structure of BcFlg was determined to provide a scheme for fusion designs. Through homology-based modeling and comparative structural analyses, diverse fusion strategies were proposed. Moreover, cellular and biophysical analysis of an array of fusion constructs indicated that insertion fusion at BcFlg residues 178–180 does not interfere with the protein stability or TLR5-stimulating capacity of flagellin, suggesting its usefulness in the development and optimization of flagellin fusion vaccines.

## Introduction

Flagella enable bacterial locomotion toward favorable conditions or away from unfavorable environments. The bacterial flagellum transverses from the cytoplasm to the outside of the bacterium and comprises more than 30 different proteins^1,2^. Among these diverse flagellar proteins, flagellin is a major component of the flagellum that forms the flagellar filament. Flagellin is produced in the cytosol and transported through the cell membrane to the cell surface, where it assembles into a filament. The flagellin polypeptide varies in length and is 249 residues in *Bacillus cereus* (BC1659) and 495 residues in *Salmonella enterica* subspecies *enterica* serovar Typhimurium (St). St flagellin (StFlg) folds into a four-domain structure that consists of the D0, D1, D2, and D3 domains^2–4^. In the flagellin filament, the D0 and D1 domains mediate inter-molecular and inter-domain interactions and form the helical stem of the filament^5^. Due to their functional importance, the D0 and D1 domains are highly conserved among flagellin orthologs^6^. In contrast, the D2 and D3 domains radiate from the D1 domain and decorate the surface of the filament. The D2 and D3 domains are hypervariable in length and amino acid sequence and are even absent in some bacterial species, including *B. cereus*.

In addition to the native function in bacteria as a structural unit of the flagellar filament, flagellin stimulates innate immunity in the host by directly interacting with Toll-like receptor 5 (TLR5), an innate immune receptor^7^. The crystal structure of a complex between zebrafish TLR5 (zfTLR5) and *S. enterica* subspecies *enterica* serovar Dublin flagellin (SdFlg) demonstrated that two flagellin molecules simultaneously bind two TLR5 receptors on the D1 domain, forming a 2:2 complex via primary and secondary contacts^8,9^. Moreover, deletional mutant studies of diverse flagellin proteins revealed that, in addition to the D1 domain, the D0 domain is required to produce a maximum TLR5-mediated response^10^.

Because flagellin is the only TLR5-specific agonist, it has been developed as a vaccine adjuvant to enhance the immunogenicity of subunit vaccines^7,11–15^. Furthermore, flagellin is a protein that can be readily fused to a protein antigen by recombinant DNA technology, and flagellin fusion proteins are currently being developed into vaccines. Fusion proteins between StFlg and antigen proteins have been experimentally evaluated to prevent diverse infectious diseases, such as cholera, influenza, West Nile fever, malaria, plague, and tuberculosis, and to treat breast and lung cancers^11,12,16–22^. Moreover, flagellin was engineered as a carrier for a cocaine vaccine to treat cocaine addiction^23^.

A flagellin fusion protein vaccine aims to simultaneously take advantage of two heterologous proteins—the flagellin and the antigen. However, the fusion process may introduce inter-molecular steric clashes or unexpected modifications, such as proteolysis or destabilization, which could reduce the efficacy of the flagellin-antigen fusion vaccine. Although flagellin has been widely applied to the development of fusion-protein vaccines, the biophysical and structural integrity of the flagellin fusion protein has never been addressed.

Because the vaccine adjuvant activity of the flagellin-antigen fusion construct is mediated by flagellin binding to TLR5 and its subsequent TLR5-activating capacity, the D0 and D1 domains of flagellin should be included for TLR5 stimulation in the flagellin-antigen fusion proteins. However, the D2 and D3 domains are not necessary for TLR5-mediated immune response and could induce an unwanted toxic immune response in the host, including in humans. Such considerations indicate that the D2 and D3 domains of flagellin should be removed from a flagellin-antigen fusion protein vaccine. Therefore, it would be useful to identify the smallest naturally occurring flagellin containing only the D0 and D1 domains. In addition, biophysical and cellular analyses of the flagellin fusion protein should be conducted to assess both the structural integrity of the fusion protein and the TLR5-stimulatory activity of the flagellin adjuvant.

In this study, *Bacillus cereus* flagellin (BcFlg) was identified as the smallest flagellin consisting only of the D0 and D1 domains that still activated innate immunity as a TLR5 agonist. Moreover, we determined the crystal structure of BcFlg at 1.85 Å resolution, revealing that the D1 domain of BcFlg adopts a well-defined helical structure. Based on comparative structural analysis of BcFlg and its orthologs and a modeling study of a complex between BcFlg and human TLR5 (hTLR5), schemes for the fusion of BcFlg with T4 lysozyme (T4L) were proposed. Through cellular and biophysical analysis of the proposed fusion constructs, we identified a flagellin fusion protein that retains its structural integrity and the TLR5-stimulatory effect of flagellin. This comprehensive study can help optimize previously proposed experimental fusion vaccines and contribute to the design of novel flagellin-antigen fusion vaccines with high protein stability.

## Results

### Flagellin from *B. cereus*

**Figure 1:**
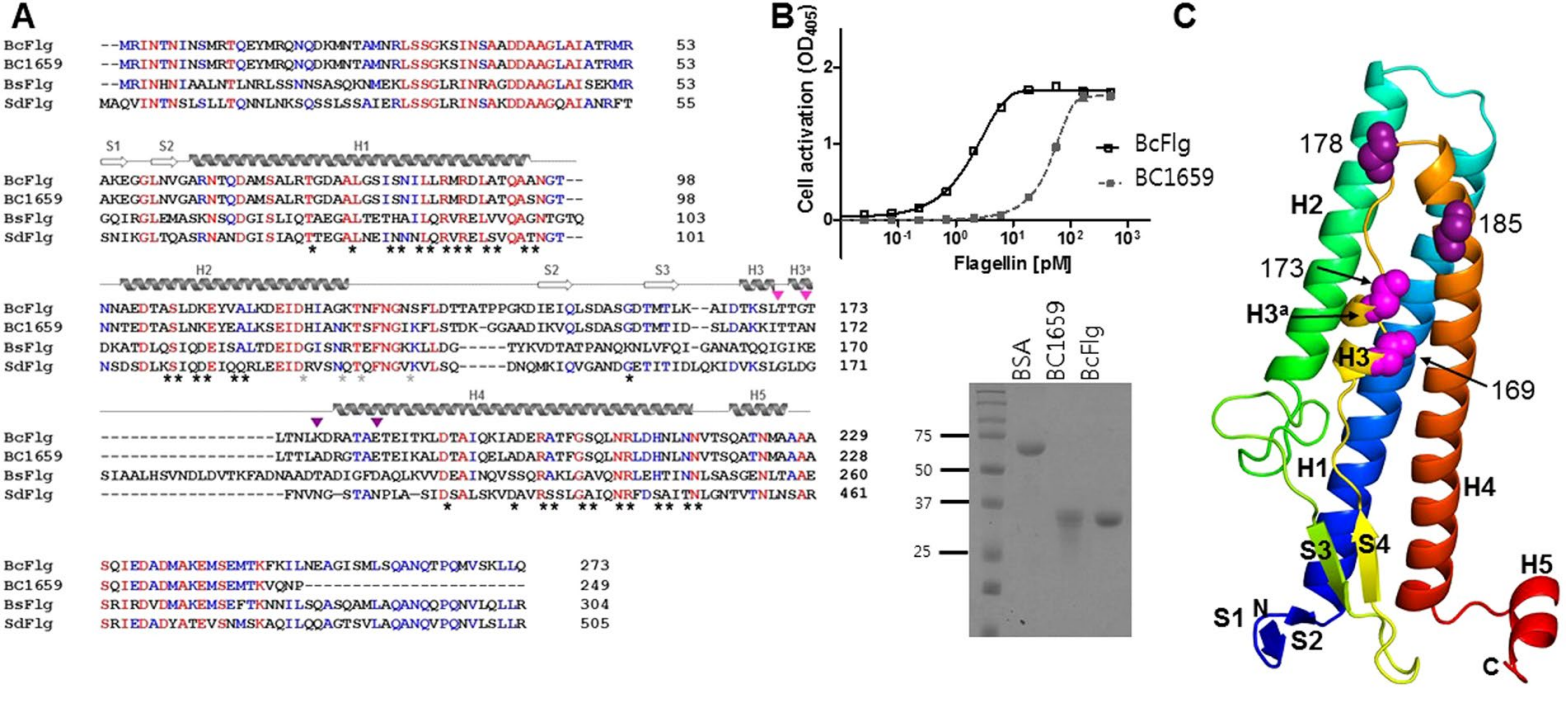
Flagellin sequences and BcFlg crystal structure. (**A**) Sequence alignments of flagellin orthologs. The secondary structures of BcFlg are shown above the BcFlg sequence. Flagellin residues involved in hTLR5 binding through 1:1 primary and 2:2 secondary interactions are highlighted by black and grey stars, respectively. The proposed insertional fusion sites are marked by magenta and purple triangles. Sequences were aligned by ClustalW^36^ and manually adjusted. (**B**) Flagellin-induced TLR5 activation analysis (top) and SDS-PAGE analysis (bottom) of BcFlg and BC1659. (**C**) Crystal structure of BcFlg. The BcFlg structure is shown in rainbow-colored ribbons. Residues located at insertional fusion sites are shown as magenta and dark magenta spheres. The N-terminus and C-terminus of the BcFlg structure are labeled ‘N’ and ‘C,’ respectively.

Because flagellin recognizes TLR5 and consequently activates innate immunity, it has been used as a fusion partner for a protein antigen in subunit vaccines to enhance vaccine efficacy^16^. Although flagellins from *Salmonella* species have been extensively studied for therapeutics and vaccines, the hypervariable D2 and D3 domains of flagellin not involved in TLR5 activation can cause unexpected toxic cellular responses *in vivo*. Thus, entolimod, an anti-radiation therapeutic drug, was designed to contain only the D0 and D1 domains of SdFlg without the D2 and D3 domains^18^. However, the artificial removal of the hypervariable domains caused protein instability and resulted in high susceptibility to proteolytic degradation. Therefore, it would be useful to employ a natural flagellin that has been evolutionarily selected to contain only TLR5-activating D0 and D1 domains and thus would be biophysically stable. Through extensive research, we identified two *B. cereus* flagellin genes, *bc1658* and *bc1659*, that contain only the D0 and D1 domains without the hypervariable domains. The BC1658 and BC1659 proteins are similar in length (BC1658, 273 residues; BC1659, 249 residues) and amino acid sequence (identity, ~84%) (Fig. 1A). Both gene products were prepared as recombinant proteins (Fig. 1B). Although both flagellin proteins were active in the TLR5-stimulation assay, BC1658 [half maximal effective concentration (EC_50_), 1.73 ± 0.10 pM] showed approximately 33-fold higher activity than BC1659 (EC_50_, 55.41 ± 1.67 pM) (Table 1). Therefore, BC1658 flagellin (BcFlg) was selected as an adjuvant agent to generate flagellin-fusion constructs.

**Table 1:** TLR5 stimulatory activities of BcFlg and its fusion proteins.

Proteins used for TLR5 stimulation	EC_50_ (pM)	Relative fold change	
BcFlg	1.7 ± 0.1	1^a^	1^b^
BcFlg_(37)_^c^	1.9 ± 0.2	N.A.^d^	1.1 ± 0.1^b^
BC1659	55.4 ± 1.7	32.6 ± 0.7^a^	N.A.^d^
BcFlg^nF^	44.2 ± 0.1	25.5 ± 3.1^a^	N.A.^d^
BcFlg^cF^	2.3 ± 0.3	1.5 ± 0.4^a^	1^b^
BcFlg^cF^_(37)_^c^	129.4 ± 19.1	N.A.^d^	74.8 ± 11.2^b^
BcFlg^mF^	1.4 ± 0.1	0.8 ± 0.7^a^	1^b^
BcFlg^mF^_(37)_^c^	2.4 ± 2.1	N.A.^d^	1.4 ± 1.2^b^

Half maximal effective concentration (EC_50_) was determined by fitting dose-dependent TLR5 activity curves using Prism (GraphPad Software). At least three independent experiments were performed in triplicate. The values represent mean ± standard deviation (SD). ^a^Relative ratio of the EC_50_ value of each protein to that of BcFlg.

^b^Relative ratio of the EC_50_ value of the protein stored after incubation at 37 °C to that of the freshly prepared one.

^c^Proteins were incubated at 37 °C for 24 hours and then used for TLR5 stimulatory experiments.

^d^N.A., not available.

### Crystal structure of BcFlg

In the design of flagellin fusion constructs, terminal fusion has been preferred to insertional fusion due to the simplicity of the design^11^. Unlike terminal fusion, insertional fusion design requires the structural information of flagellin to prevent plausible steric clashes between a target protein and flagellin, which would reduce the efficacy of vaccines. To provide a schematic plan for insertional fusion sites, the crystal structure of BcFlg protein was determined at 1.85 Å resolution (Fig. 1C, Supplementary Table S1). The structure contains BcFlg residues 54–229 corresponding to the D1 domain, which was shown to directly interact with TLR5 in a previously reported SdFlg-zfTLR5 structure. Thus, the BcFlg structure can be used to identify possible insertional fusion sites that would not interfere with TLR5 binding. The BcFlg structure presents a topology of S1 (residues 54–56) - S2 (residues 59–61) - H1 (residues 63–98) - H2 (residues 104–127) - S3 (residues 147–150) - S4 (residues 158–161) - H3 (residues 166–169) - H3^a^ (residues 171–173) - H4 (residues 180–216) - H5 (residues 221–226) (Figs 1A,C and S1). The H1, H2, H4, and H5 helices are α-helices, and the H3 and H3^a^ helices adopt 3_10_ helical structures.

**Figure 2:**
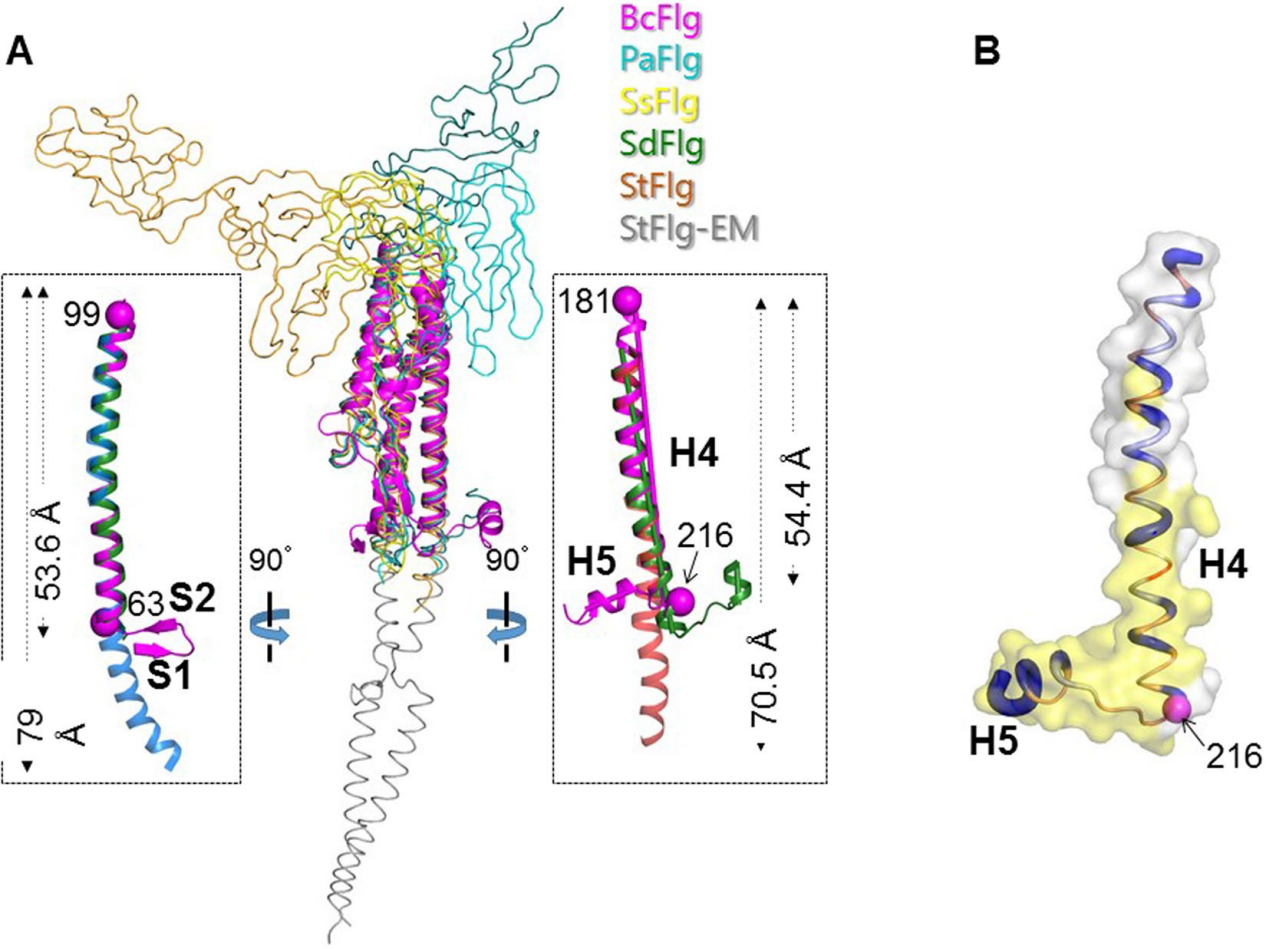
Comparative structural analysis of the BcFlg and its orthologs. (**A**) Overlays of the BcFlg structure (magenta ribbons) and other flagellin structures (coils) are shown [PaFlg (PDB ID 4NX9, RMSD of 1.35 Å and sequence identities of 33.0% for 176 residues, cyan), SsFlg (PDB ID 2ZBI, RMSD with 1.46 Å and sequence identities of 32.0% for 176 residues, yellow), SdFlg (PDB ID 3V47, RMSD with 1.51 Å and sequence identities of 34.0% for 176 residues, green), and StFlg (PDB ID 1IO1, RMSD with 2.70 Å and sequence identities of 9.6%, for 176 residues, orange)]. The N-terminal and C-terminal regions of BcFlg adopted different conformations from  those of its orthologs and are shown in ribbons in the left and right boxes, respectively. The lengths and angles of α-helices were calculated using the Chimera program. The helical axes for C-terminal helices of BcFlg and SdFlg are shown as magenta and green rods, respectively, and were used to calculate inter-helix angles. (**B**) The C-terminal helices of BcFlg are shown as surface presentation and coils. Coils are red, white, or blue according to the helical propensity of each residue in a range of 0–1. The BcFlg residues that are modeled to interact with TLR5 are highlighted by yellow surfaces. BcFlg residue 216 at the C-terminal end of the H4 helix is represented by a magenta sphere.

BcFlg is structurally similar to its orthologs (PaFlg, SsFlg, SdFlg, and StFlg) in that it contains a three-helix bundle structure (H1, H2, and H4) appended to an S3-S4 β-hairpin (Fig. 2A)^3,4,6,8,24^. In contrast to the common fold, the BcFlg structure is distinguishable from other flagellin structures in forming unique conformations at the N-terminal and C-terminal regions (Figs 1C and 2A). BcFlg residues 54–61 that correspond to the N-terminal region of the D1 domain fold into two β-strands, S1 and S2, and their connecting loop. However, in the crystal structures of other flagellin proteins, the region equivalent to BcFlg residues 54–61 was not structurally defined, presumably due to its high structural flexibility or proteolytic degradation during sample preparation. In the EM structure of the StFlg filament, the corresponding region was resolved to adopt an α-helix that belongs to the H1 helix with a helical length of 79 Å (Fig. 2A, left inset). This comparative analysis of various flagellin structures suggests that the N-terminal region of the D1 domain is structurally diverse and flexible.

The C-terminal region of the flagellin D1 domain also exhibited structural diversity, although it generally retains a helical propensity (Fig. 2B). In the EM structure of the StFlg filament^4^, the C-terminal region of the D1 domain forms a 70.5-Å-long helix. In contrast, in the BcFlg structure, the C-terminal helix in the D1 domain is split into two α-helices, H4 and H5, which are distanced by 11.3 Å and angled by 72.9°. In the SdFlg-zfTLR5 complex structure, the C-terminal region of SdFlg D1 is also discontinued to two α-helices as for the BcFlg structure but with a different distance (16.9 Å) and angle (13.8°). These observations suggest that the C-terminal region of the D1 domain is a pliable helix that can bend and adopt diverse conformations.

### Structure-based design of insertional fusion sites

**Figure 3:**
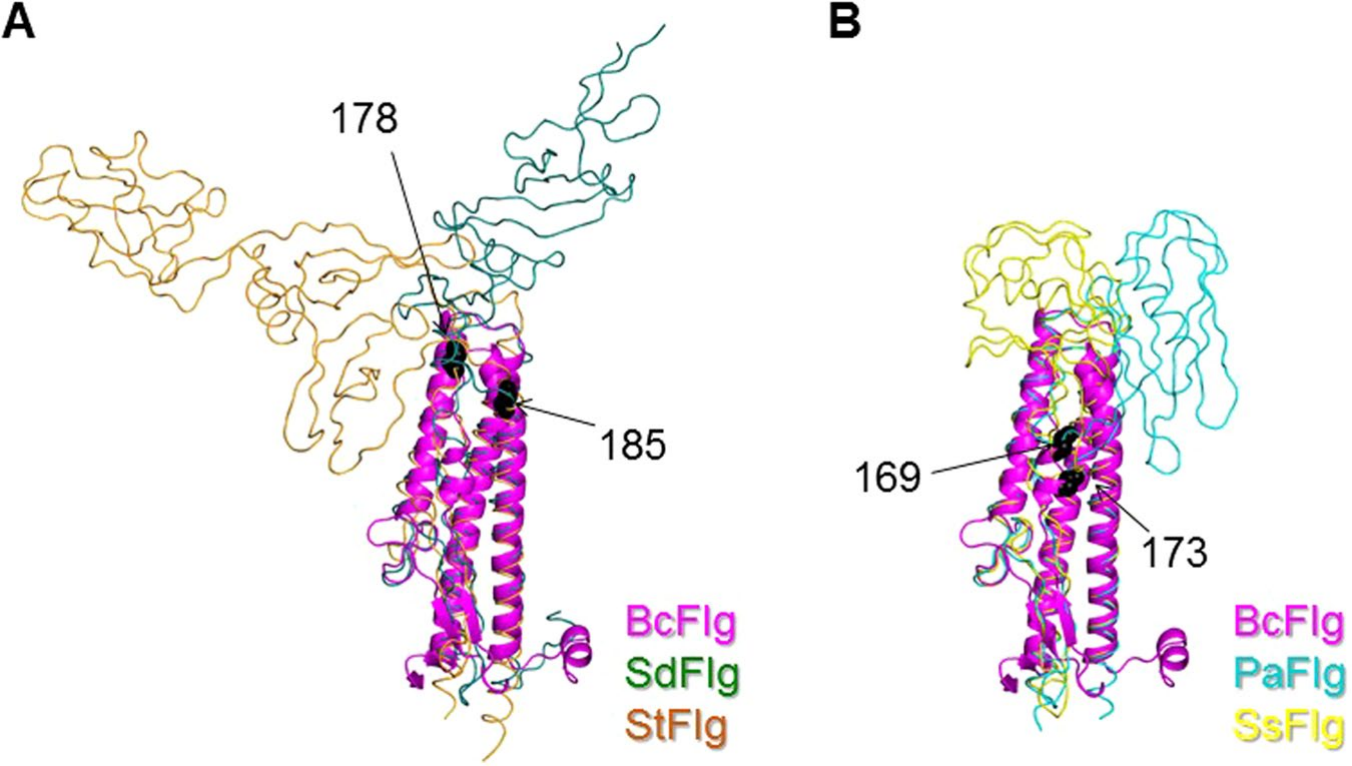
Insertional fusion sites mFa (BcFlg residues 178 and 185: (**A**) and mF (BcFlg residues 169 and 173: (**B**) used to generate the BcFlg^mF^ and BcFlg^mFa^ fusion proteins, respectively. The fusion sites are black in the BcFlg structure (magenta ribbons), which is overlaid with SdFlg (dark green coils) and StFlg (orange coils) structure (**A**) and with the PaFlg (cyan coils) and SsFlg (yellow coils) structure (**B**).

In the design of flagellin-antigen fusion proteins, the fusion site where a target antigen is ligated with the flagellin polypeptide can be selected via comparative structural analysis of BcFlg and other multi-domain flagellin proteins containing hypervariable domains. The rationale for the insertional fusion design is homology-based replacement, in which the fusion protein mimics a natural multi-domain flagellin protein by replacing the hypervariable domains of flagellin with a target antigen. In multi-domain flagellin proteins, insertion of hypervariable domains into the D1 domain has been likely optimized through an evolutionary process. In a previous report, a chimeric flagellin was generated by inserting the hypervariable D2 and D3 domains of *Helicobacter pylori* flagellin into the middle of the D1 domain of *Escherichia coli* flagellin and was shown to stimulate immune response against *H. pylori*^25^. Thus, in combinations of BcFlg and the target antigen, the fusion protein can be designed to mimic a natural multi-domain flagellin protein and will be expected not to destroy the structural integrity of the fusion protein. In the structural overlays of flagellin proteins using the D1 domain, two possible replacement sites were located where hypervariable domains are appended to the D1 domain of flagellin. In the SdFlg and StFlg structures, the hypervariable domains are linked to a point corresponding to BcFlg residues 178–185 (Fig. 3A). Because the residues 181–185 form an α helix, an insertional site was selected in BcFlg residues 178–180 (middle fusion, mF) so as not to interrupt the secondary structural fold. In the PaFlg and SsFlg structures, the D2 domain extends from a loop corresponding to BcFlg residues 169–173 (additional middle fusion, mFa) (Fig. 3B). We chose the mF and mFa sites for generation of insertional fusion proteins.

### Structure model of the BcFlg-hTLR5 complex to validate the TLR5-binding activity of flagellin fusion proteins

**Figure 4:**
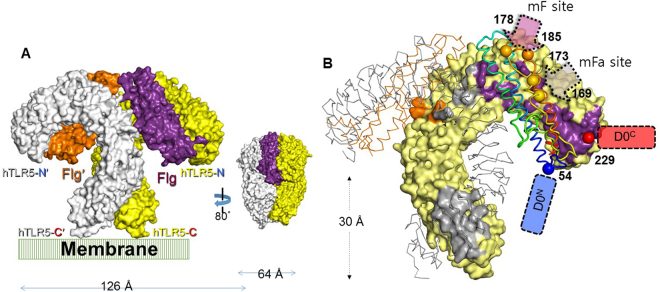
Homology-based structural model of the BcFlg-hTLR5 complex. (**A**) Two BcFlg chains (BcFlg and BcFlg′, purple and orange surfaces, respectively) and two hTLR5 chains (hTLR5 and hTLR5′, yellow and grey surfaces, respectively) constitute the BcFlg-hTLR5 complex. The cell membrane is schematically shown below the BcFlg-hTLR5 complex. The N- and C-terminal ends of the hTLR5 extracellular domain are labeled hTLR5-N and hTLR5-C, respectively. Dimensions of the quaternary BcFlg-hTLR5 complex are indicated. (**B**) Interactions of the hTLR5 chain with the BcFlg, BcFlg′, and hTLR5′ chains. In the 1:1 BcFlg:hTLR5 heterocomplex, 23 BcFlg residues make contact with 44 hTLR5 residues (intermolecular distance cutoff, 4 Å). In the 2:2 homodimer between BcFlg:hTLR5 and BcFlg′:hTLR5′ heterodimers, four BcFlg residues are expected to interact with five hTLR5′ residues. The BcFlg and BcFlg′ chains are represented by rainbow-colored coils and orange lines, respectively. The hTLR5 and hTLR5′ chains are shown with a yellow surface and grey line, respectively. TLR5 residues that make contact with BcFlg, BcFlg′, and hTLR5′ are highlighted in purple, orange, and grey, respectively, on the TLR5 surface. The N-terminal and C-terminal regions of the BcFlg D0 domain are schematically depicted as light blue and orange blocks, respectively. BcFlg residues at insertional fusion sites mF and mFa are labeled and indicated by arrows. The N-terminal residue 54 and C-terminal residue 229 of the BcFlg D1 domain structure are indicated by red and blue spheres, respectively.

**Figure 5:**
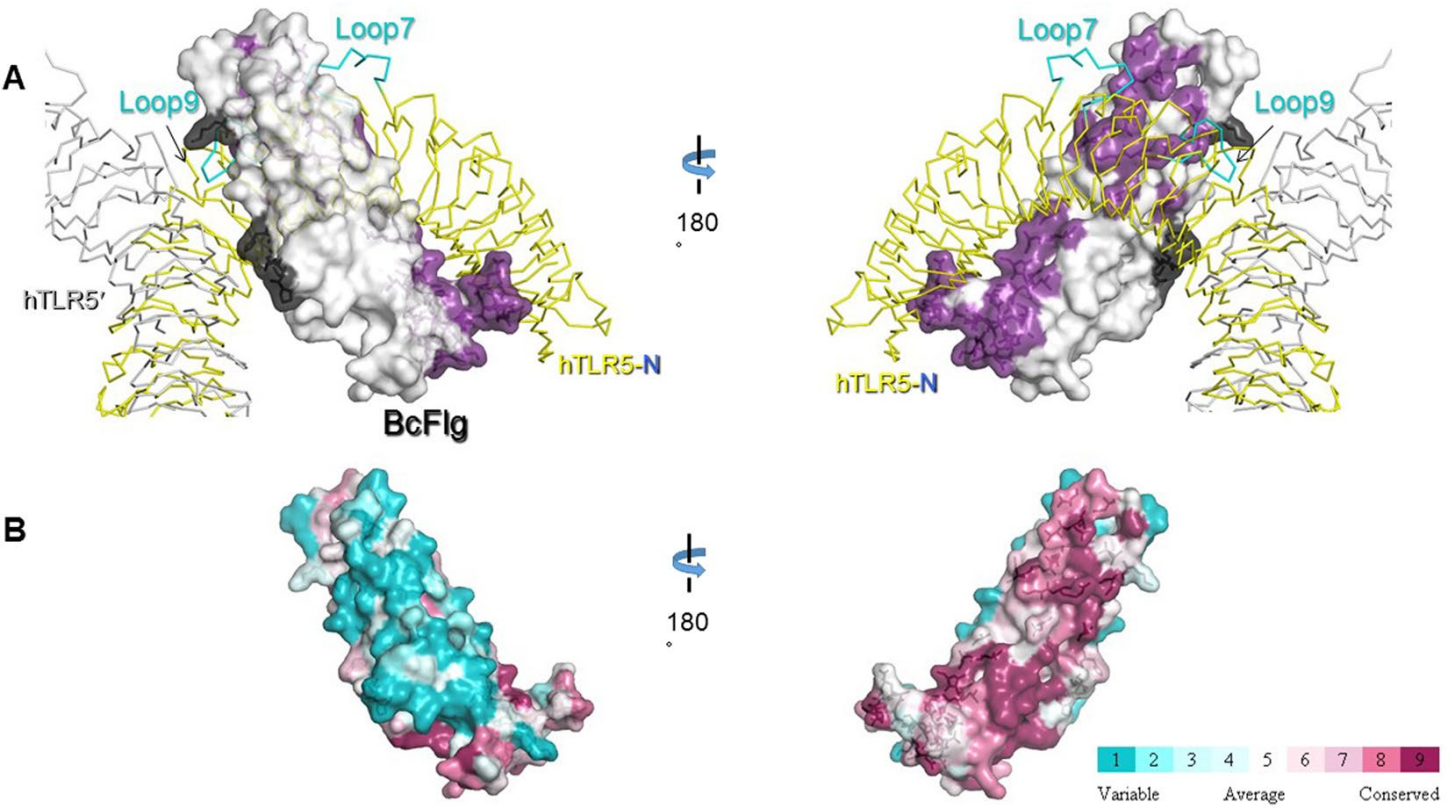
TLR5-binding residues of BcFlg and their sequence conservation. (**A**) BcFlg residues involved in hTLR5 and hTLR5′ binding are colored by purple and dark grey, respectively, on the light grey surface of BcFlg. hTLR5 and hTLR5′ are shown as yellow and grey lines, respectively. The TLR5-specific loops at LRR7 and LRR9 (labeled ‘Loop7′ and ‘Loop9’, respectively) are shown as cyan lines. (**B**) Sequence conservation of the flagellin D1 domain is color-coded from cyan (low conservation) to red (high conservation).

In addition to retaining the structural integrity of flagellin, the flagellin-fusion construct should be a functional agonist of TLR5 for an application in vaccine design. To ensure that the flagellin-fusion protein is able to form an active complex with TLR5, we performed *in silico* structural analysis of a complex formation between BcFlg and human TLR5 (hTLR5). Although the SdFlg-zfTLR5 complex structure has provided invaluable information regarding flagellin recognition by TLR5, a few questions remain unresolved. First, because TLR5 in the complex structure was derived from zebrafish, not humans, the applicability of the structure to human diseases is limited. Second, the zfTLR5 structure displays only the N-terminal region of zfTLR5, corresponding to approximately 60% of the entire extracellular domain of zfTLR5. The remaining C-terminal region of the TLR5 extracellular domain has not been structurally defined, although it is critical to constrain the formation of the flagellin-TLR5 complex. Third, the D0 domain of flagellin is required to maximize TLR5-mediated cell response, but was not located in the SdFlg-zfTLR5 structure. Thus, we generated a complete structural model of the BcFlg-hTLR5 complex through comparative structural overlays and homology-based modeling (Fig. S2)^8,26,27^. In the BcFlg-hTLR5 complex model, two copies of BcFlg and two copies of hTLR5 assemble into a dimer of a heterodimer [BcFlg:hTLR5 and BcFlg′:hTLR5′ (the prime symbol denotes the dimerization partner)], and the C-terminal regions of two hTLR5 chains gather in the middle of the complex (Fig. 4). The hTLR5-binding and hTLR5′-binding residues of BcFlg are highly conserved throughout the flagellin orthologs, suggesting that the BcFlg:hTLR5 complex model is reliable (Fig. 5).

It has not yet been experimentally determined where the D0 domain of BcFlg is positioned in complex with TLR5. The D0 domain is disordered in the monomeric state, but forms α-helices in the flagellar filament, which can be observed in the EM structure of the StFlg filament^4,28–30^. In the StFlg EM structure, the D0 domain consists of two longitudinal α-helices and forms an elongated straight rod together with the D1 domain. Thus, when the EM structure of StFlg is overlaid on the BcFlg-hTLR5 complex model using the flagellin D1 domain, the D0 domain of StFlg experiences substantial steric clashes with the cell membrane, suggesting that the straight rod of the D0 and D1 domains in the StFlg filament is not compatible with TLR5 binding (Fig. S3). Interestingly, unlike the EM structure of StFlg, the N-terminal and C-terminal regions of the BcFlg D1 domain that correspond to D0-D1 boundary regions are bent in different directions from the main axis of the D1 rod (Fig. 4B). If the conformations of the terminal regions of the BcFlg D1 domain are maintained in the flagellin-TLR5 structure, the C-terminal part of the BcFlg D0 domain can be traced outward from the flagellin-TLR5 complex, and the N-terminal region of the BcFlg D0 domain occurs along the C-terminal part of the TLR5 extracellular domain without clashes with the cell membrane. Taken together, the conformational flexibility of the D0 domain and the D0-D1 boundary region of flagellin facilitates the fusion of a target antigen at the N-terminal or C-terminal end of BcFlg and prevents interference in interactions between flagellin and membrane-anchored TLR5.

The BcFlg:hTLR5 complex model also positively supports the idea that fusion at the proposed mF and mFa sites does not structurally interfere with the formation of the BcFlg-hTLR5 complex (Fig. 4B). The mF and mFa sites are located at solvent-exposed loops on the opposite side of the TLR5-binding interface of BcFlg and on the top of the 2:2 complex, respectively. When T4 lysozyme (T4L) was located at the proposed fusion sites of the BcFlg:hTLR5 complex, no major steric crashes with BcFlg and TLR5 were observed (Fig. S3). Therefore, the insertional fusion of a target antigen into BcFlg using the mF and mFa sites would be structurally acceptable in complex formation with hTLR5.

### Validation of BcFlg-target fusion designs through cellular and biophysical analyses

**Figure 6:**
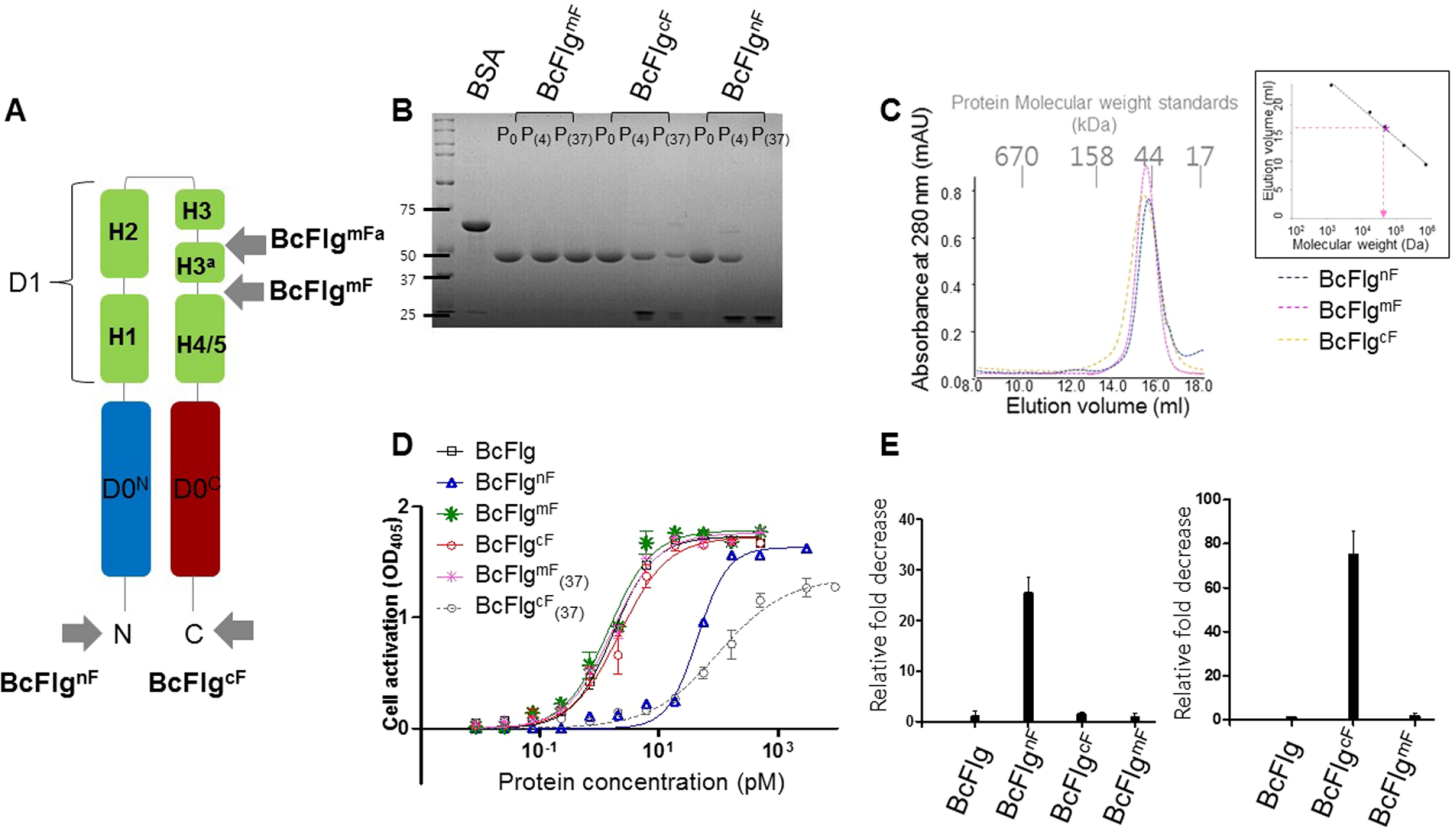
Biophysical and cellular analyses of the BcFlg-T4L fusion constructs. (**A**) Schematic representations of the BcFlg-T4L fusion designs. The N-terminal and C-terminal segments of the D0 domain are represented by light blue and red rods, respectively. Each helix in the D1 domain is shown as a green rod. The insertion sites of T4L in BcFlg are indicated by arrows. (**B**) SDS-PAGE analysis of BcFlg-T4L fusion proteins. In each lane, 4 μg of fusion protein (P_0_, freshly made; P_(4)_, stored at 4 °C for 24 hours; P_(37)_, stored at 37 °C for 24 hours) was loaded. (**C**) Gel-filtration chromatography analysis of the recombinant BcFlg^mF^ protein. Using the elution volumes of gel-filtration standards (vertical lines in the inset), the apparent molecular weight of the BcFlg-T4L fusion protein was estimated to be ~50 kDa, similarly to the calculated molecular weight of ~49.4 kDa. The elution profile of BcFlg^mF^ is similar to other soluble fusion proteins (BcFlg^nF^ and BcFlg^cF^). (**D**) TLR5-stimulating activities of BcFlg and BcFlg-T4L fusion proteins. The subscript ‘(37)’ indicates that the protein was stored at 37 °C for 24 hours and then analyzed. (**E**) Relative fold decreases of the TLR5-stimulating activities of the BcFlg-T4L fusion proteins compared to that of BcFlg (left). The chart shows the representative data from at least three independent experiments that yielded similar results. Relative fold decrease of the TLR5-stimulating activity of 37 °C-stored BcFlg or BcFlg-T4L fusion proteins compared to that of the freshly prepared counterpart (right).

To empirically analyze any changes in the structural integrity and in TLR5-activating ability of flagellin upon fusion with a target protein, we produced four BcFlg fusion proteins. For linear fusion, T4L was appended to the N-terminal and C-terminal ends of BcFlg to generate BcFlg^nF^ and BcFlg^cF^ fusion constructs, respectively (Figs 6A, S1B). For insertional fusion, T4L was inserted into BcFlg residues 169–173 and residues 178–180 to generate BcFlg^mFa^ and BcFlg^mF^ fusion constructs, respectively (Figs 6A, S1B). The BcFlg^nF^, BcFlg^cF^, and BcFlg^mF^ fusion proteins were expressed as soluble proteins (Fig. 6B) and purified as monomers based on gel-filtration chromatography analysis (estimated molecular weight, ~50 kDa; calculated molecular weight, ~49.4 kDa) (Fig. 6C). However, BcFlg^mFa^ was expressed as an insoluble protein presumably because the mFa site is closely located to flagellin core helices H2 and H4 (an approximately 5 Å distance between the Cα carbon atoms of H2 residue 121 and mFa residue 172; an approximately 7 Å distance between the Cα carbon atoms of mFa residue 170 and H4 residue 193) and would disrupt the helical bundle structure of the BcFlg (Fig. S3A). Thus, the BcFlg^mFa^ fusion protein was excluded from further analysis.

The TLR5-activating capacity of the BcFlg^nF^, BcFlg^cF^, and BcFlg^mF^ fusion proteins was analyzed using a TLR5 reporter cell assay. BcFlg^cF^ and BcFlg^mF^ stimulated TLR5-induced innate immunity in a comparable manner to BcFlg (Table 1). However, BcFlg^nF^ displayed an approximately 26-fold lower TLR5 stimulatory activity than that of the BcFlg, potentially due to protein instability (Table 1 and Fig. 6B,D,E). The BcFlg^nF^ fusion protein underwent substantial proteolytic degradation when stored at 4 °C and 37 °C for 48 hours (Fig. 6B). As for BcFlg^nF^, BcFlg^cF^ was also vulnerable to proteolytic degradation, and 37 °C-incubated BcFlg^cF^ protein exhibited approximately 75-fold lower TLR5 stimulatory activity compared to that of freshly prepared BcFlg^cF^ protein (Table 1 and Fig. 6D,E). In contrast to terminal fusions, such as in BcFlg^nF^ and BcFlg^cF^, the insertional fusion protein BcFlg^mF^ was fully functional at stimulating a TLR5 response, and its TLR5-stimulting activity was not diminished even with 37 °C storage (Table 1 and Fig. 6D,E). Moreover, the 37 °C-stored BcFlg^mF^ protein did not produce any proteolytically truncated fragments in SDS-PAGE analysis (Fig. 6B). Collectively, our comprehensive structural, biophysical, and cellular analyses enabled us to identify an optimized insertional flagellin-fusion design that retains both protein integrity of the fusion protein and TLR5-stimulating activity of flagellin.

## Conclusion

An antigen-adjuvant fusion vaccine has the advantage of simultaneously delivering both antigen and adjuvant to tissue. As a vaccine fusion partner, flagellin has been widely studied because it specifically activates TLR5-mediated innate immunity as a vaccine adjuvant and is easily conjugated to a proteinaceous antigen in an upstream process by recombinant technology. In previously designed fusion protein vaccines, fusion was performed without structural consideration and biophysical characterization. Indeed, flagellin fusion proteins in which T4L was linked to the N-terminal or C-terminal end of BcFlg were vulnerable to proteolysis. In this study, structural homology-based replacement was used to select insertional fusion sites in flagellin, and the fusion sites were experimentally analyzed through cellular and biophysical analyses. Based on these comprehensive studies, we conclude that insertional fusion at BcFlg residues 178–180 does not interfere with the protein stability or the TLR5-stimulating activity of flagellin and can be used to design future flagellin-conjugated vaccines.

## Methods

### Preparation of recombinant BcFlg protein and its fusion proteins

To generate *B. cereus* flagellin-expression vectors, the two flagellin-encoding genes (*bc1658* gene, residues 1–273; *bc1659* gene, residues 1–249) were amplified by polymerase chain reaction (PCR) from the genomic DNA of *B. cereus* ATCC 14579 using primers that contain *Bam*HI or *Sal*I restriction-enzyme sites. The PCR product was digested using *Bam*HI and *Sal*I and ligated into a modified pET49b vector that contains an N-terminal His_6_ tag and a thrombin cleavage site^31^. The ligation product was used to transform *E. coli* DH5α cells, and the nucleotide sequence of transformants was confirmed by DNA sequencing.

DNA fragments that encode the fusion proteins of BcFlg and T4 lysozyme (T4L) were generated by overlapping PCR and inserted into the modified pET49b by enzyme digestion and ligation. The identities of the fusion constructs were verified by DNA sequencing.

Flagellin proteins [BcFlg (BC1658) and BC1659] and T4L-fused BcFlg proteins were overexpressed in *E. coli* strain BL21 (DE3) in the presence of 1 mM isopropyl β-D-1-thiogalactopyranoside at 37 °C for approximately 4 h. Cells were pelleted by centrifugation and lysed by sonication in phosphate-buffered saline (PBS, pH 7.4) containing 10 mM imidazole. The cell lysate was centrifuged (approximately 25,000 × g) to remove insoluble precipitates. The cleared cell lysate that contained soluble protein was incubated with Ni-NTA agarose resin (Qiagen). The His_6_-tagged protein was competitively eluted using 250 mM imidazole in PBS and dialyzed against 50 mM Tris, pH 7.4, and 50 mM NaCl.

### Crystallization and X-ray diffraction data collection

For crystallization, the D1 domain of BcFlg that contained residues 52–230 was expressed and purified by Ni-NTA chromatography, as for BcFlg. The BcFlg D1 domain was treated with thrombin to remove the N-terminal His_6_ affinity tag. The tag-free protein was further purified by anion-exchange chromatography using a Mono Q 10/100 GL column (GE Healthcare). The BcFlg protein was injected into the column equilibrated in 50 mM Tris, pH 7.4 and was eluted from the column by a linear NaCl gradient. Based on the SDS-PAGE analysis of fractions, BcFlg protein was eluted at a conductivity of approximately 9.2 mS · cm^−1^. The resulting BcFlg protein was concentrated to 16.1 mg/ml for crystallization.

The crystallization conditions of BcFlg were screened by the sitting-drop vapor-diffusion method at 18 °C. BcFlg crystals were obtained with 0.1 M glycine, pH 10, 0.2 M lithium sulfate, 1.2 M sodium dihydrogen phosphate, and 0.8 M di-potassium hydrogen phosphate. For X-ray diffraction data collection, BcFlg crystals were cryoprotected in a crystallization solution containing 30% glycerol. A single crystal was flash-frozen under a cryo-stream at −173 °C. X-ray diffraction was performed at beamline 7 A of the Pohang Accelerator Laboratory (PAL, Pohang, Korea). Diffraction data were indexed, integrated, and scaled using the HKL2000 package^32^. X-ray diffraction statistics are shown in Supplementary Table S1.

### Structure determination of BcFlg

The phases of the crystal structure of the BcFlg D1 domain were calculated by molecular replacement using a partial structure (residues 56–139 and 307–331) of *Sphingomonas* species flagellin (SsFlg, PDB ID 3K8W)^24^ as a search model by Phaser^33^. The final structure of BcFlg was obtained by iterative model building and refinement procedures using the Coot^34^ and Refmac5^35^ programs, respectively. Structural refinement statistics are listed in Table 1.

### Determination of protein concentration and integrity

Because flagellin protein does not contain any tryptophan, tyrosine, or cysteine residues that absorb 280-nm light, the concentration of flagellin protein used in this study was determined by Bradford assay (Biorad) using a standard curve of bovine serum albumin. The calculated protein concentration was confirmed by the intensity of Coomassie brilliant blue-stained bands for 4 μg of protein in SDS-PAGE gels before a cell-based TLR5-stimulation assay. SDS-PAGE analysis was also used to confirm the concentration and the integrity of the protein.

### Flagellin-induced hTLR5 stimulation assay

Flagellin-induced TLR5 activation was assessed using the HEK293blue-hTLR5 reporter cell line (InvivoGen). The HEK293blue-hTLR5 cell contains a gene that encodes secreted embryonic alkaline phosphatase (SEAP), which is inducible by TLR5-mediated NF-κB activation. The assay was performed as described in the manufacturer’s protocol^10^. Briefly, HEK293blue-hTLR5 cells were maintained in DMEM-high glucose supplemented with 10% fetal bovine serum, 4.5 mg/ml glucose, 2 mM L-glutamine, 15 μg/ml blasticidin, and 100 μg/ml zeocin. For the flagellin-induced TLR5 activation assay, 80 μl of 4 × 10^5^ HEK293blue-hTLR5 cells were prepared in each well of 96-well plates. Then, 20 μl of flagellin or its fusion proteins were serially diluted and added into each well to stimulate TLR5. After 12 hours of ligand treatment, the amount of secreted alkaline phosphatase was measured by incubating with 100 μl of an alkaline phosphatase substrate, *p*-nitrophenyl phosphate (Sigma-Aldrich). Then, the reaction was diluted five-fold in 20 mM Hepes, pH 7.4 and 150 mM NaCl and analyzed for the formation of colorimetric product using a spectrophotometer at a wavelength of 405 nm.

### Data availability

The atomic coordinates and structure factors for BcFlg (PDB ID 5Z7Q) have been deposited in the Protein Data Bank, www.pdb.org.

## Electronic supplementary material

Supplementary information
