# Supplementary material for: Crystal structure of Bacillus cereus flagellin and structure-guided fusion-protein designs
Source: Sci Rep. 2018 Apr 11;8:5814. doi: 10.1038/s41598-018-24254-w (PMC5895748; doi:10.1038/s41598-018-24254-w)
Supplement: Supplementary file 1 — Supplementary information [file 41598_2018_24254_MOESM1_ESM.pdf]

# Crystal structure of *Bacillus cereus* flagellin and structure-guided fusion-protein designs

Meong Il Kim[1], Choongdeok Lee[1], Jaewan Park[1], Bo-Young Jeon[2], and Minsun Hong[1*]

[1]Division of Biological Science and Technology, Yonsei University, Wonju 26493, Republic of Korea

[2]Department of Biomedical Laboratory Science, Yonsei University, Wonju 26493 Republic of Korea.

*To whom correspondence should be addressed.

- Minsun Hong (E-mail: minsunhong@yonsei.ac.kr, Phone: +82-33-760-2243, Fax: +82-33-760-2183)

Supplementary Table S1. Crystallographic statistics of the BcFlg structure.

| | **BcFlg** |
|---|---|
| **Data collection** | |
| Space group | $P6_2$ |
| Cell parameters | a = 112.72 Å |
| | b = 112.72 Å |
| | c = 40.19 Å |
| Wavelength (Å) | 1.00002 |
| Resolution (Å) | 32.72 - 1.85 |
| Highest resolution (Å) | 1.88 - 1.85 |
| No. of observations | 138,340 |
| No. of unique reflections | 48,450 |
| $R_{merge}$ (%)[a] | 6.6 (28.1)[b] |
| I/sigma | 14.4 (3.3)[b] |
| Completeness (%) | 99.6 (98.9)[b] |
| Redundancy | 5.5 (3.9)[b] |
| **Refinement** | |
| Resolution (Å) | 20.00 - 1.85 |
| No. of reflections (total) | 25,229 |
| No. of reflections (test) | 1,218 |
| $R_{cryst}$ (%)[c] | 18.6 |
| $R_{free}$ (%)[d] | 19.6 |
| Average B-value ($Å^2$) | 27.2 |
| No. of protein atoms | 847 |
| No. of water molecules | 153 |
| RMSD bonds (Å) | 0.009 |
| RMSD angles (°) | 1.163 |
| Ramachandran[e] (favored) | 99.0% |
| (outliers) | 0.0 % |

[a]$R_{merge} = \Sigma_{hkl}\Sigma_i \mid I_i(hkl) - \langle I(hkl)\rangle \mid / \Sigma_{hkl}\Sigma_i I_i(hkl)$

[b]Numbers in parentheses were calculated from data of the highest resolution shell.

[c]$R_{cryst} = \Sigma \| F_{obs}|-|F_{calc}\| / \Sigma|F_{obs}|$, where $F_{calc}$ and $F_{obs}$ are the calculated and observed structural factor amplitudes, respectively

[d]$R_{free}$ = as for $R_{cryst}$, but for 5.06% of the total reflections chosen at random and omitted from refinement

[e]Calculated using MolProbity [16].

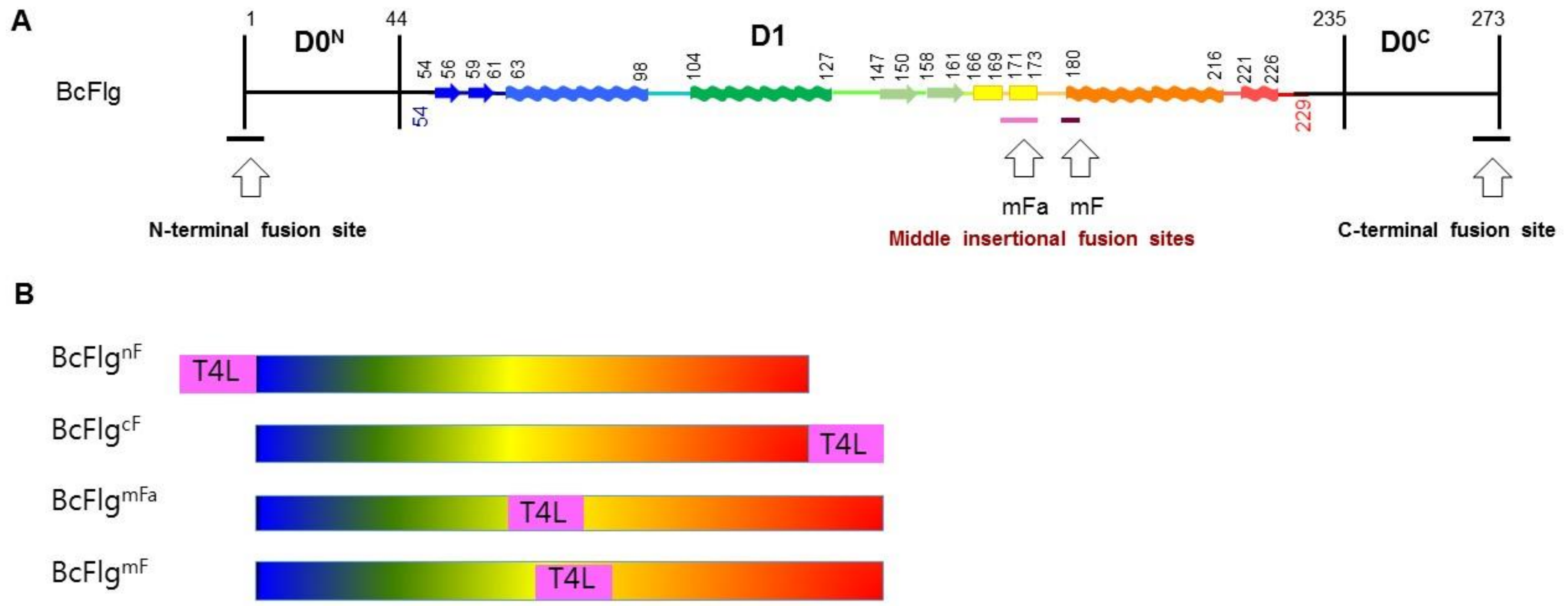


**Supplementary Figure S1**. The schematic representation of BcFlg and BcFlg-T4L fusion proteins. (A) Schematic figure of the D0 and D1 domains of BcFlg. Fusion sites are indicated by arrows with labels. (B) Fusion constructs generated in this study.

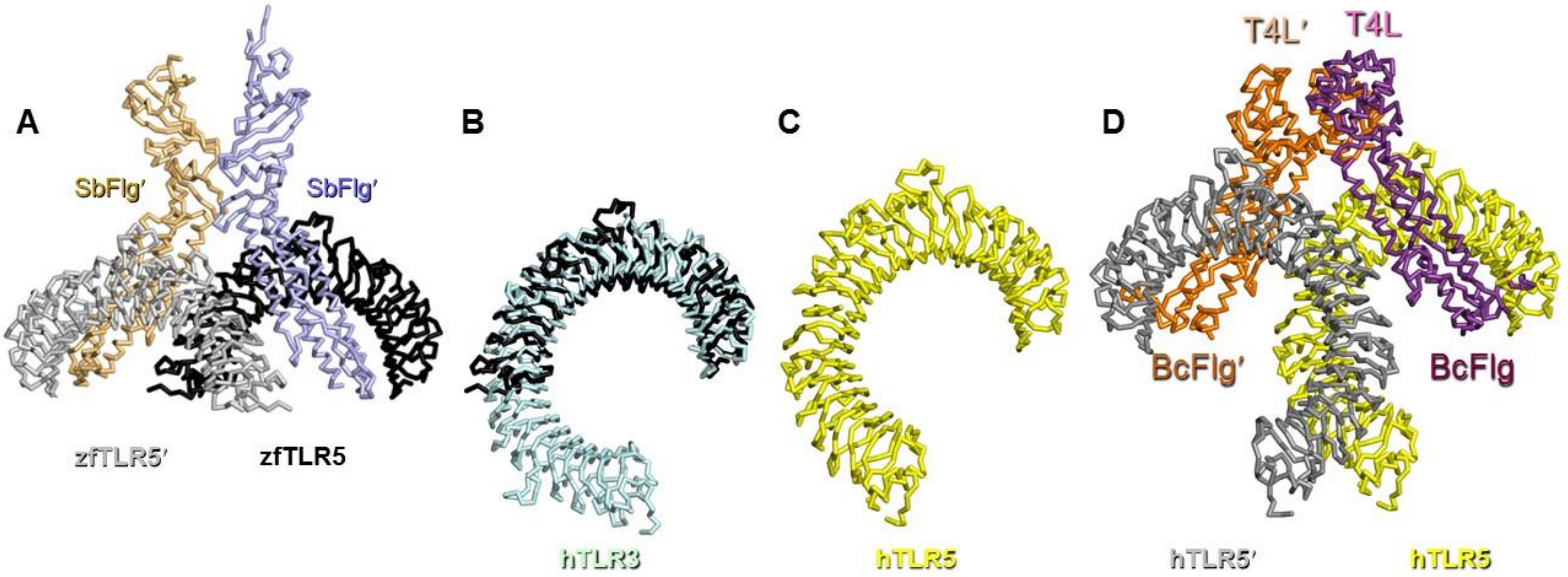


**Supplementary Figure S2**. Homology-based structural modeling of the BcFlg$^{mF}$-hTLR5 complex. (A) Crystal structure of a complex between SdFlg (orange and light blue) and zfTLR5 (black and grey). (B) Structural overlays of zfTLR5 (black) and hTLR3 (light cyan, PDB ID 2A0Z). (C) Homology model of hTLR5 (yellow). The complete extracellular domain structure of hTLR5 was generated by homology-based modeling. First, the zfTLR5 structure was overlaid on the hTLR3 structure as shown in Fig. S2B. Next, structures corresponding to zfTLR5 residues 1-397 and hTLR3 residues 460-696 were combined to build the entire extracellular structural model of the entire extracellular domain (residues 19-633) of TLR5. (D) Structural model of a complex between BcFlg$^{mF}$ (BcFlg, magenta and orange; T4L, light magenta and light orange) and hTLR5 (yellow and grey). To build the structural model of the 2:2 BcFlg$^{mF}$:hTLR5 complex, the BcFlg structure and the hTLR5 model were overlaid with SdFlg and zfTLR5, respectively, in the SdFlg-zfTLR5 complex structure. T4L was located by replacing the D2 domain of SdFlg in the SdFlg-zfTLR5 structure. The resulting BcFlg$^{mF}$- hTLR5 model was energy-minimized using the Refmac5 program to yield the final model.

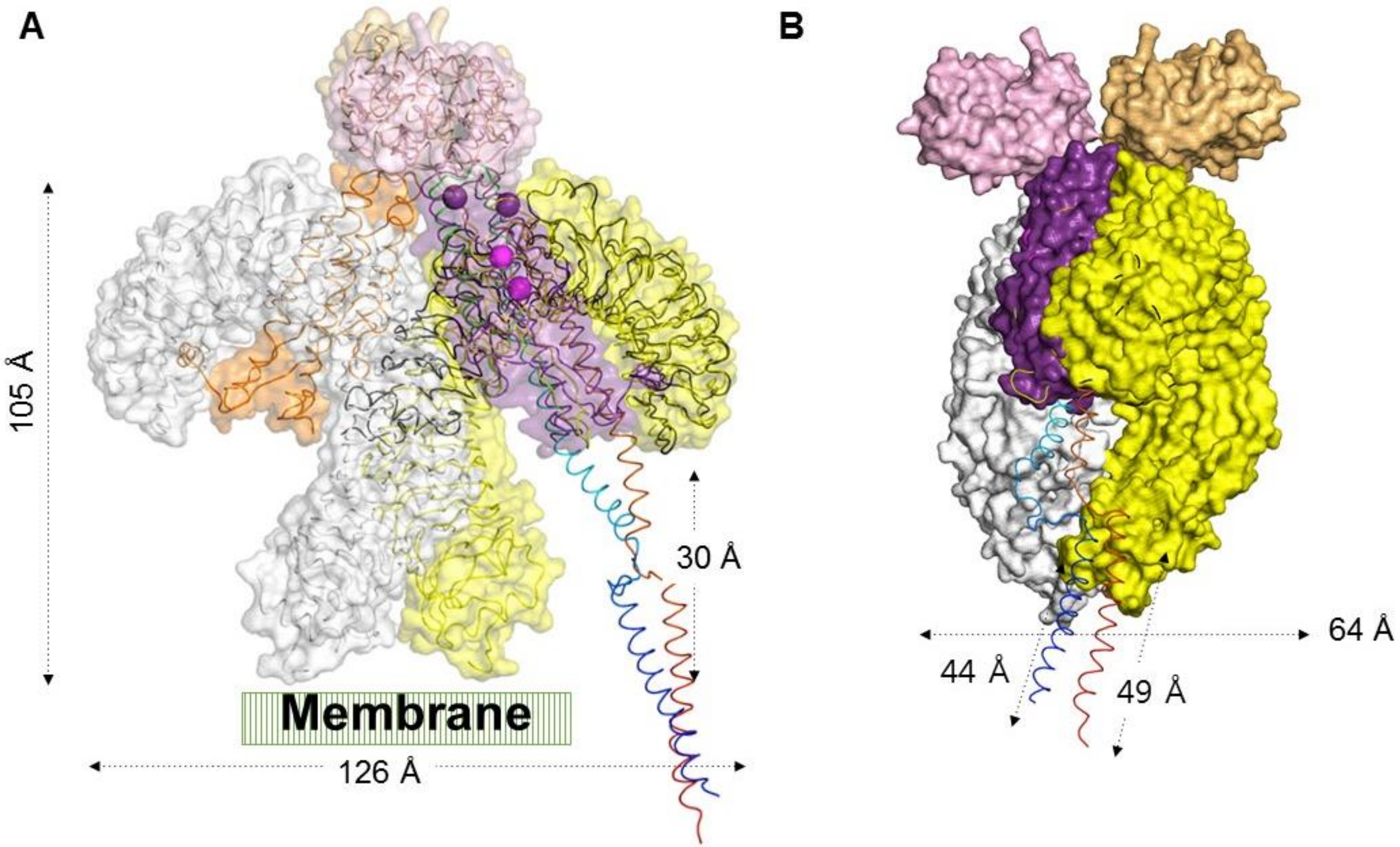


**Supplementary Figure S3**. Implausible conformation of the D0 domain of the StFlg EM structure when StFlg forms a complex with TLR5 on the cell membrane. The EM structure of StFlg (rainbow-colored coils, PDB ID 1UCU) was superimposed on the BcFlg chain of the BcFlg$^{mF}$-hTLR5 complex model (BcFlg, magenta and orange surfaces; T4L, light magenta and light orange surfaces; hTLR5, yellow and grey surfaces) to show that the D0 domain of StFlg produces steric clashes with the cell membrane. The dimensions of the quaternary complex are indicated. Fig. S3B was generated by rotating Fig. S3A by 80° along the y-axis
